# Supplementary material for: A gene desert required for regulatory control of pleiotropic Shox2 expression and embryonic survival
Source: Nat Commun. 2024 Oct 10;15:8793. doi: 10.1038/s41467-024-53009-7 (PMC11467299; doi:10.1038/s41467-024-53009-7)
Supplement: Supplementary file 1 — Supplementary Information [file 41467_2024_53009_MOESM1_ESM.pdf]

SUPPLEMENTARY INFORMATION

A gene desert required for regulatory control of pleiotropic *Shox2* expression and embryonic survival

Samuel Abassah-Oppong[1,13,#], Matteo Zoia[2,#], Brandon J. Mannion[3,4,#], Raquel Rouco[5], Virginie Tissières[2,6,7], Cailyn H. Spurrell[3], Virginia Roland[2], Fabrice Darbellay[3,5], Anja Itum[1], Julie Gamart[2,7], Tabitha A. Festa-Daroux[1], Carly S. Sullivan[1], Michael Kosicki[3], Eddie Rodríguez-Carballo[8,14], Yoko Fukuda-Yuzawa[3], Riana D. Hunter[3], Catherine S. Novak[3], Ingrid Plajzer-Frick[3], Stella Tran[3], Jennifer A. Akiyama[3], Diane E. Dickel[3], Javier Lopez-Rios[6,9], Iros Barozzi[3,10], Guillaume Andrey[5], Axel Visel[3,11,12], Len A. Pennacchio[3,4,11], John Cobb[1,*] and Marco Osterwalder[2,3,7*]

1 Department of Biological Sciences, University of Calgary, 2500 University Drive N.W., Calgary, Alberta, T2N 1N4, Canada.

2 Department for BioMedical Research (DBMR), University of Bern, 3008 Bern, Switzerland.

3 Environmental Genomics and Systems Biology Division, Lawrence Berkeley National Laboratory, Berkeley, CA 94720, USA.

4 Comparative Biochemistry Program, University of California, Berkeley, CA 94720, USA.

5 Department of Genetic Medicine and Development and iGE3, Faculty of Medicine, University of Geneva, 1211 Geneva, Switzerland.

6 Centro Andaluz de Biología del Desarrollo (CABD), CSIC-Universidad Pablo de Olavide-Junta de Andalucía, 41013 Seville, Spain.

7 Department of Cardiology, Bern University Hospital, 3010 Bern, Switzerland.

8 Department of Genetics and Evolution, University of Geneva, 1211 Geneva, Switzerland.

9 School of Health Sciences, Universidad Loyola Andalucía, Seville, Spain.

10 Center for Cancer Research, Medical University of Vienna, Vienna, Austria.

11 US Department of Energy Joint Genome Institute, Lawrence Berkeley National Laboratory, Berkeley, CA 94720, USA.

12 School of Natural Sciences, University of California, Merced, Merced, CA 95343, USA.

13 Present address: Department of Biological Sciences, Fort Hays State University, Hays, KS 67601, USA.

14 Present address: Department of Molecular Biology, University of Geneva, 1211 Geneva, Switzerland.

# These authors contributed equally.

* Correspondence: jacobb@calgary.ca (J.C.), marco.osterwalder@unibe.ch (M.O.)

Supplementary Figures

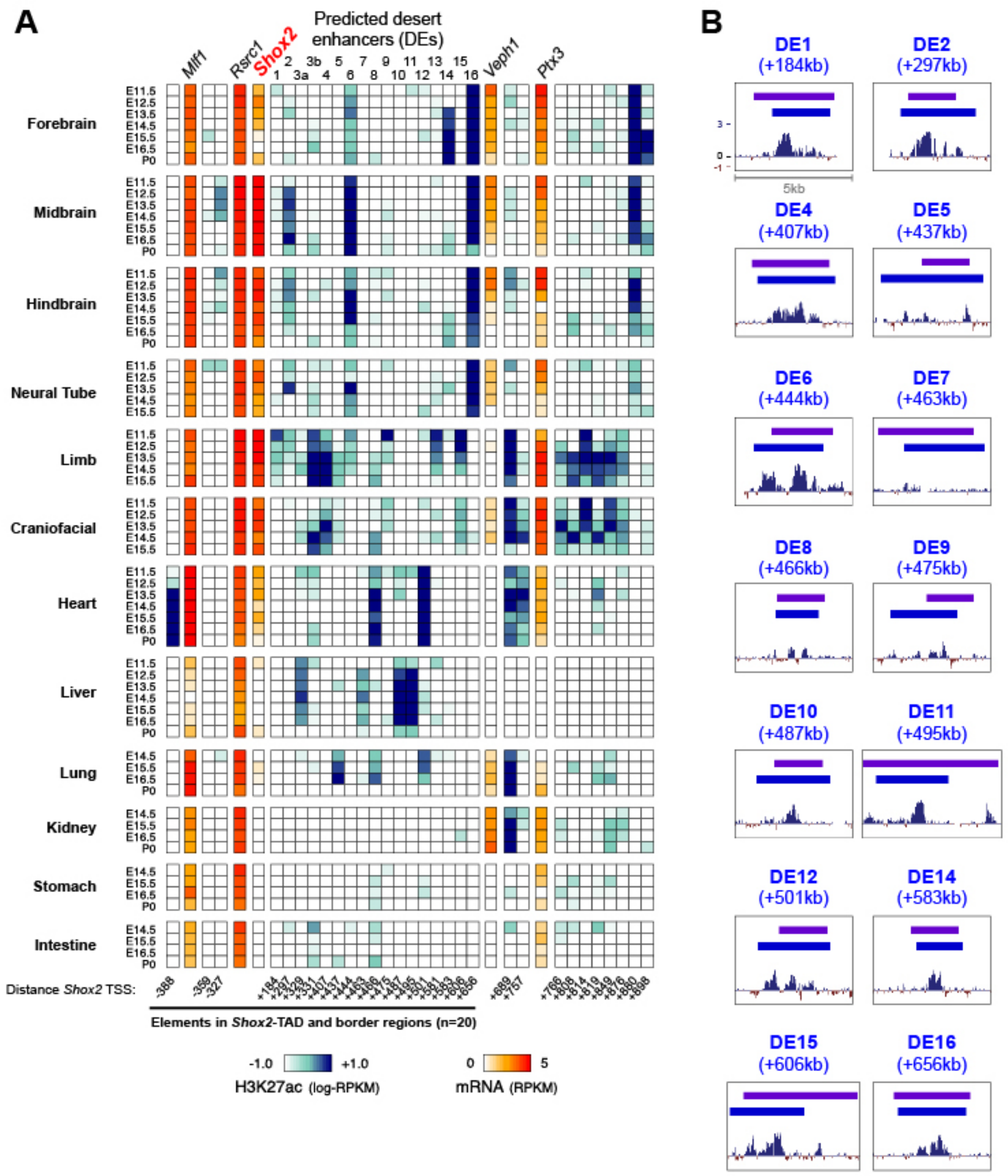

Supplementary Figure 1. Spatio-temporal H3K27ac signatures and conservation of gene desert enhancers (DEs) at the *Shox2* locus. (**A**) Heat map for prediction of tissue-specific enhancers within the extended *Shox2* TAD[1] across different time points and tissues, based on H3K27ac marks[2] (ENCODE) and ChromHMM filtering (see Methods). Blue shading indicates levels of H3K27ac ChIP-seq enrichment and red shades illustrate transcript levels (ENCODE RNA-seq datasets) of genes located in the region (Supplementary Data 1). Predicted gene desert enhancer (DE) IDs are listed on top with distance (in kb) to *Shox2* TSS in the bottom (-, upstream; +, downstream). (**B**) Base-wise conservation track by PhyloP (placental mammals) for each DE with validated tissue-specific enhancer activity in mouse embryos at E11.5 (Fig. 1C).

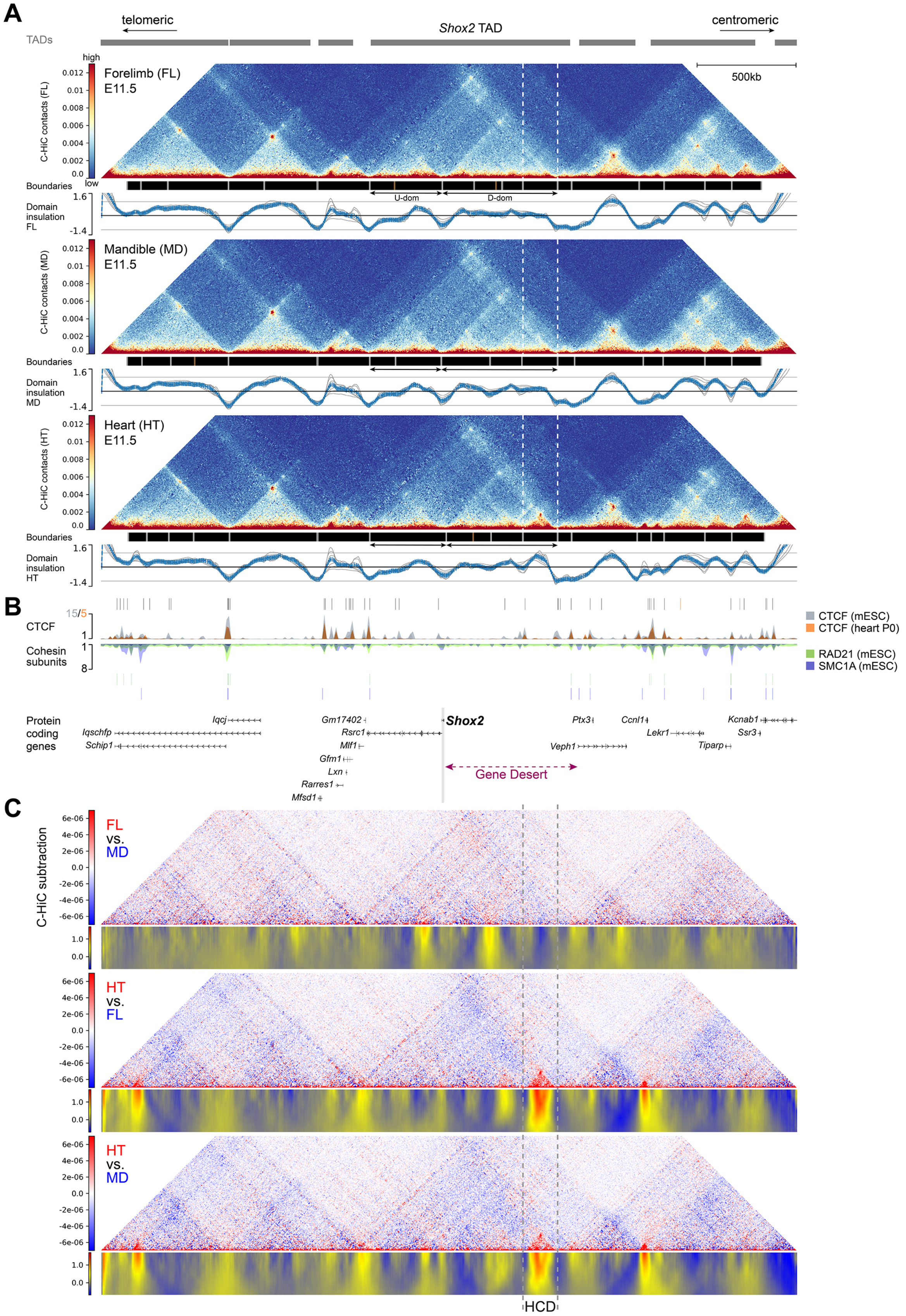

Supplementary Figure 2. 3D chromatin interactions across the extended *Shox2* genomic locus. (**A**) Chromatin conformation across the 3.5Mb C-HiC probe interval (mm10, chr3:65196078-68696078) centered on the *Shox2* TAD from forelimb (FL), mandibular process (MD) and heart (HT) tissues of mouse wildtype embryos at E11.5. Top: C-HiC matrices revealing 3D chromatin contacts.

Middle: Gray boxes (with white frames) on black background represent domain boundaries according to significant TAD separation score ($p<0.01$). Brown boxes represent weaker boundaries ($p<0.05$). Bottom: Plot depicting normalized inter-domain insulation scores (lowest values correspond to strongest insulation). TAD intervals from mouse embryonic stem cells (mESCs) are represented as black bars on top[3]. *Shox2* U-dom (upstream domain) and D-Dom (downstream domain) are indicated by double arrows. (**B**) Top: CTCF profiles and peak calls in mESCs[3] (gray) and mouse heart at P0[4] (orange). Middle: Profiles and peak calls of SMC1A and RAD21 cohesin subunits in mESCs[5]. Bottom: The extension of the *Shox2* gene desert is indicated (purple double arrow) along with protein coding genes. (**C**) Top: Subtraction of C-HiC profiles in pairwise tissue comparisons (red/blue) reveals a heart-specific high contact chromatin domain (HCD, see also in Fig. 2). Bottom: Matrices depicting subtraction of normalized inter-domain insulation scores based on pairwise tissue comparisons above. Strong interaction (red) is observed across the HCD interval in HT vs. FL and HT vs. MD, but not FL vs. MD comparisons.

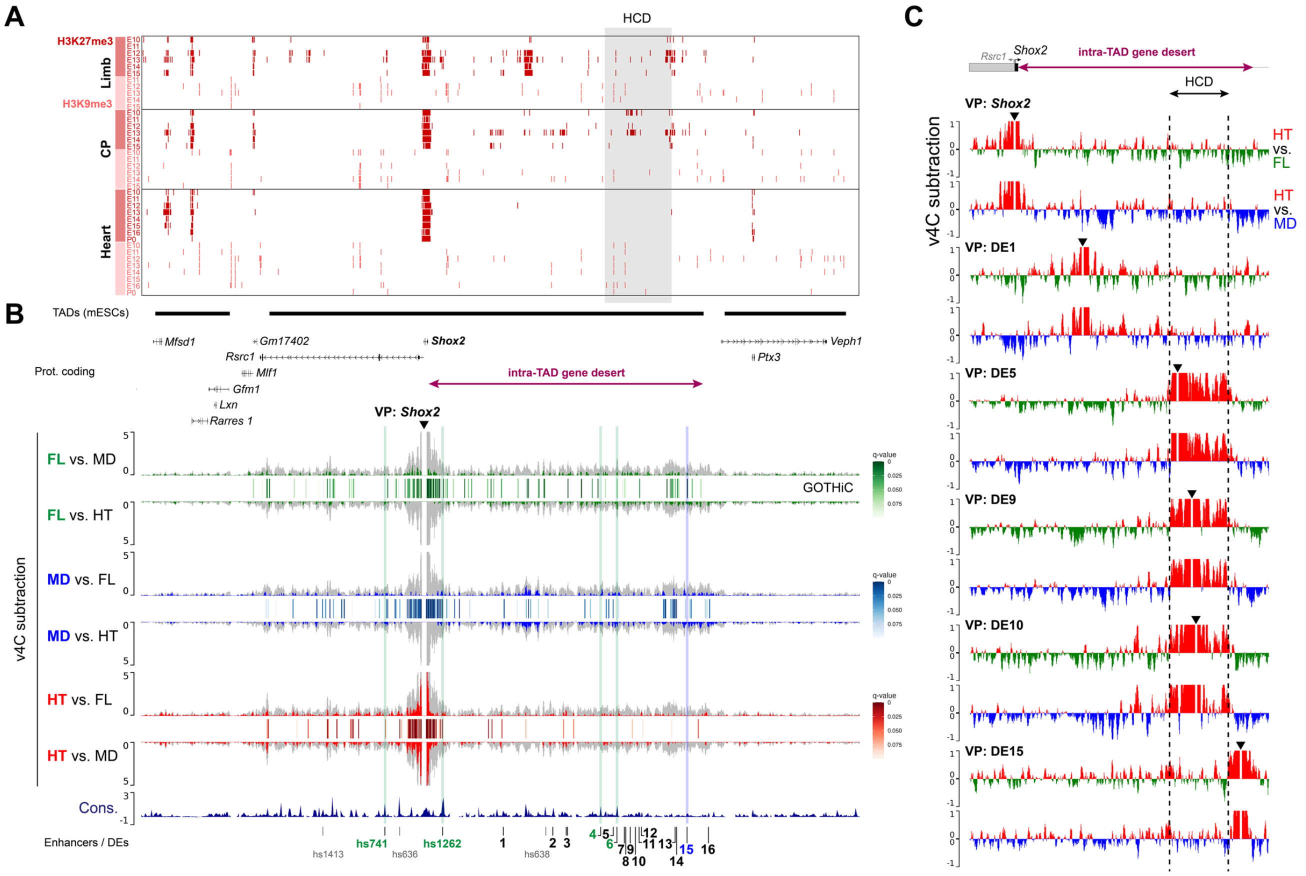

Supplementary Figure 3. Distribution of repressive histone marks and *Shox2* promoter contacts across the extended *Shox2* TAD. (**A**) Distribution of ENCODE ChIP-seq peak calls from histone modification marks associated with repressive chromatin functions (H3K27me3, H3K9me3) in limb (E10.5-E15.5), craniofacial prominence (CP) (E10.5-E15.5) and heart tissues (E10.5-E16.5, P0)[2]. The high-density contact domain (HCD) interval (Fig. 2, Supplementary Fig. 2) is shaded in gray. (**B**) Region-wide *Shox2* contacts based on virtual 4C (v4C) analysis from C-HiC in forelimb (FL), mandible (MD) and heart (HT) across the extended *Shox2* TAD (chr3:65977711-67631930) are shown in gray (see also Fig. 2A). V4C subtraction analysis indicates tissue-enriched contacts in pairwise comparisons (green: FL, blue: MD, red: HT). Bars between tissue-related profiles represent *Shox2* contact regions corrected for bias and based on a significance threshold as identified by GOTHiC[6]. Validated enhancers with activities in the FL or MD are indicated by green and blue lines, respectively. TAD extensions from mESCs are shown[3]. Cons, Placental Conservation by PhyloP. 1-16, DEs. (**C**) V4C subtraction profiles of HT vs. FL and HT vs. MD comparisons using different VPs across the intra-TAD gene desert. VPs include *Shox2* and selected DEs (3kb intervals). In forelimb (green peaks) and mandible (blue peaks) the enhancer VPs within the HCD (DE5, 9 and 10) display numerous contacts with regions outside of the HCD, while in the heart (red peaks) tissue-enriched contacts from the HCD with outside regions appear reduced. VPs located outside of the HCD in the heart (DE 1, 15) show more distributed contacts.

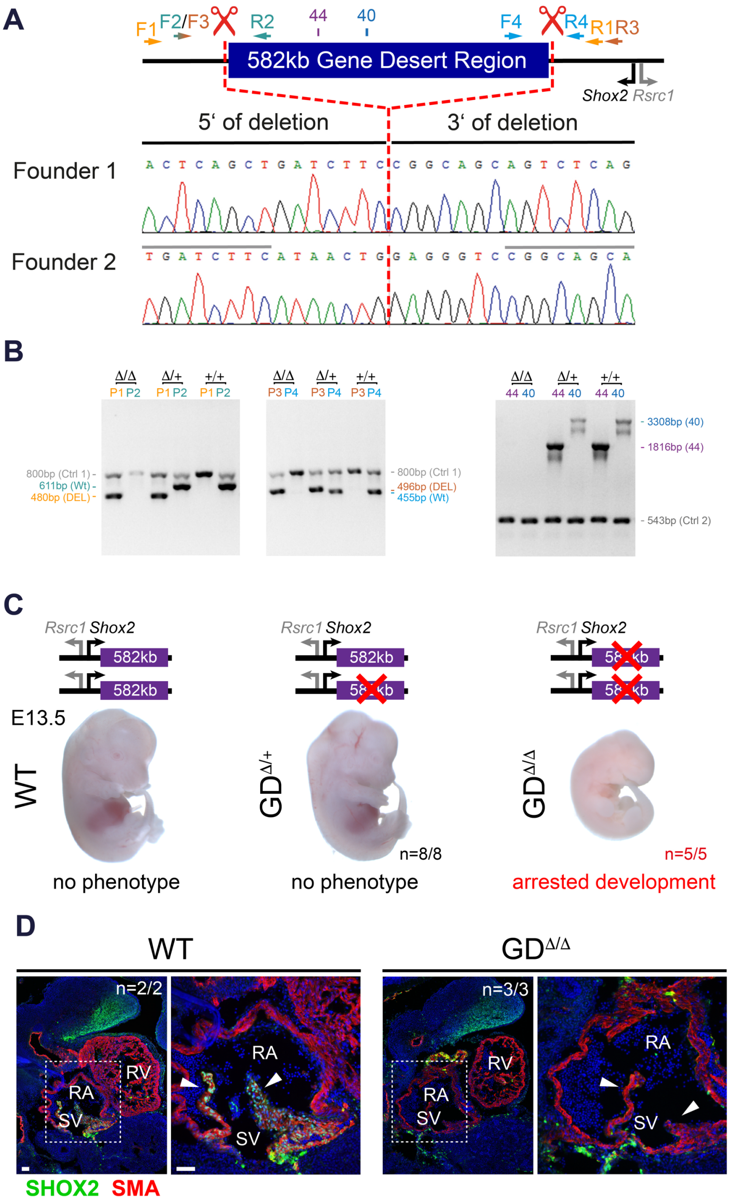

Supplementary Figure 4. Gene desert deletion triggers embryonic lethality linked to loss of *Shox2* in the SV. (**A**) Sanger sequencing traces revealing clean gene desert deletion breakpoints (dashed red line) in the mouse lines generated via CRISPR-Cas9 for this study. Red scissors indicate the location of the guide RNAs used (Supplementary Table 2). Location of primers (arrows) and amplicons (blocks) used for PCR are indicated (Supplementary Table 3). (**B**) PCR validation and genotyping used to detect wildtype (+) and GD$^{\Delta}$ (DEL) alleles. Amplicon sizes are indicated on the side. Control primers (Ctrl 1 or Ctrl 2) amplifying an unrelated genomic region were used. P, primer pair (+ or DEL) mixed with control primers. (**C**) Homozygous gene desert deletion (GD$^{\Delta/\Delta}$) leads to arrested mouse embryonic development past E11.5, with full penetrance at E13.5, recapitulating the pattern observed in *Shox2*-deficient embryos[7]. Heterozygous (GD$^{\Delta/+}$) genotypes develop into viable and fertile mice. (**D**) SHOX2 (green) is depleted in myocardial cells (SMA, red) of the SV including venous valves (white arrowheads) in GD$^{\Delta/\Delta}$ embryos at E11.5. Nuclei are stained blue. Scale bars, 50µm. RA, right atrium. RV, right ventricle. SV, sinus venosus. N, number of biological replicates with similar results.

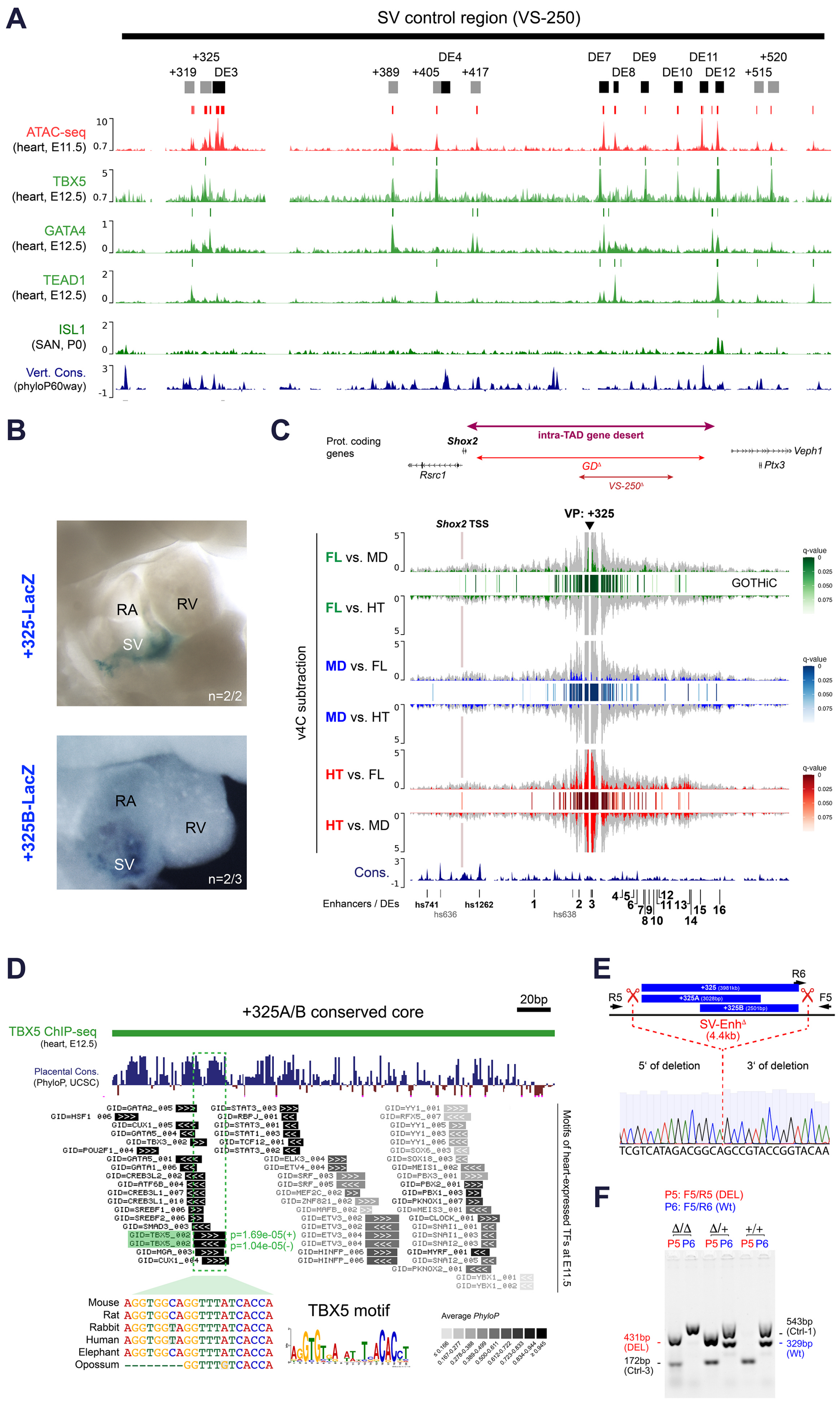

Supplementary Figure 5. TF binding profile and cardiac specificity of the +325 SV enhancer and its genomic deletion in mice. (**A**) Reprocessed ChIP-seq profiles from cardiac TFs involved in SAN specification[8–10] (green) in the sinus venosus (SV) control region (VS-250)[11]. Open chromatin profiles (ATAC-seq) and vertebrate conservation track are shown for comparison. Peak calls are indicated on top of each track. Genomic elements validated in this study are displayed on top (see

also Fig. 4D). (**B**) Transgenic LacZ reporter validation of the +325 element (3981bp) and the +325B subregion (2501bp) at E11.5. N indicates transgenic replicates with similar staining. RA, right atrium. RV, right ventricle. (**C**) Region-wide v4C analysis and contacts from C-HiC in forelimb (FL), mandible (MD) and heart (HT) using the conserved +325A interval (chr3:66656000-66660000) as viewpoint (analogous to Supplementary Fig. 3B). Tissue-enriched contacts in pairwise comparisons (green: FL, blue: MD, red: HT) and GOTHiC-predicted regions contacting the +325 element (Supplementary Data 2) are indicated. (**D**) Cardiac TF motif analysis (see Methods) reveals a TBX5 motif within the +325A/B conserved core region overlapping local TBX5 enrichment in hearts at E12.5[8]. This motif is conserved across mammals and matches a TBX5 dimer DNA binding domain[12]. Cons., mammalian conservation by PhyloP (UCSC browser). All motifs annotated match a *p*-value of $\leq 10^{-4}$ (FIMO). *P*-values correspond to the probability of a random sequence (of the same length as the motif) producing a score that matches at least the one observed. Corresponding gene IDs (GID) are listed for each motif. (**E**) Sanger sequencing results of the deletion breakpoint in the +325 SV enhancer knockout allele (SV-Enh$^{\Delta}$) generated using CRISPR-EZ in mouse zygotes[13] (see Methods). The location of primers used for PCR genotyping is indicated (Supplementary Table 3). (**F**) PCR primer pairs (P5 and P6) to genotype the SV-Enh$^{\Delta}$ (Δ) and wildtype (+) alleles were mixed with Ctrl-3 and Ctrl-1 primer pairs, respectively, to allow for amplification of a control band in the same PCR reaction.

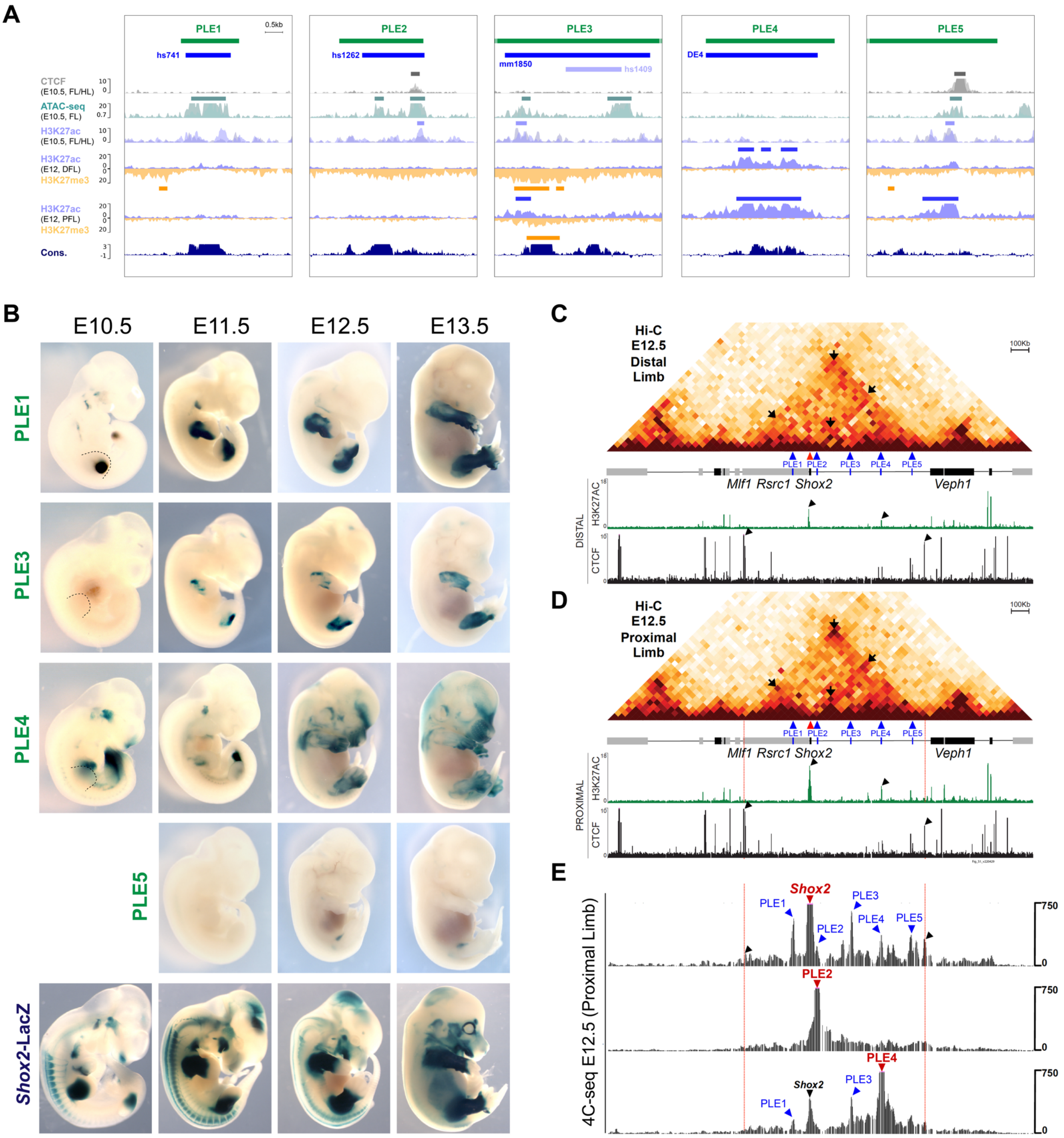

Supplementary Figure 6. Spatiotemporal activities of *Shox2*-PLEs and associated 3D-chromatin interactions. (**A**) Chromatin signatures of PLEs (green bars, see also **Supplementary Table 7**) in mouse embryonic limbs at E10.5 (CTCF, ATAC-seq, H3K27ac) and E12 (H3K27ac, H3K27me3), as shown locus-wide in Fig. 6A. Blue bars on top represent previously validated limb enhancers (PLE1, PLE2)[14,15] or elements with reproducible limb enhancer activities confirmed in this study (DE4/mm1850), as listed in the Vista Enhancer Browser. Bars above each track represent peak calls across replicates. DFL, distal forelimb. PFL, proximal forelimb. (**B**) Developmental time course of proximal limb enhancer (PLE) activities in stable transgenic LacZ reporter lines compared to *Shox2* expression (*Shox2*-LacZ). For each element, one embryo from one representative transgenic line is shown per time point (Supplementary Table 7 *and* Methods). To aid in visualization, embryos are depicted at progressively lower magnification at later stages. Dashed line indicates forelimb bud in PLE embryos at E10.5. (**C, D**) Comparison of chromatin interactions within a 2.4Mb region centering the *Shox2* locus (mm10, chr3:65720000-68120000) in distal (C) and

proximal (D) limbs at E12.5, including Hi-C, H3K27Ac and CTCF ChIP-seq tracks from a previous study[16]. *Shox2*-TAD boundaries are delineated by CTCF peaks (black arrowheads) and marked by red lines. Hi-C and H3K27ac profiles indicate that several interactions are stronger in proximal limb cells as compared to those in distal limb progenitors. Of the four highlighted interaction points (black arrows in the Hi-C map), two indicate *Shox2* interaction with U-dom and D-dom anchors (left and right arrows), while the upper arrow represents a strong interaction between TAD anchor points. The bottom arrow indicates a stronger interaction of *Shox2* with PLE3 in the proximal limb. Genes are shown as rectangles, with black indicating genes transcribed from left to right (telomeric to centromeric) and gray indicating genes transcribed in the opposite direction. (**E**) 4C-seq interaction profiles with PLE2 (hs1262/LHB-A) and PLE4 as viewpoints (purple arrowheads), compared to the profile based on the *Shox2* viewpoint (Fig. 6B, Supplementary Table 6). One of two biologically independent replicates with similar results is shown.

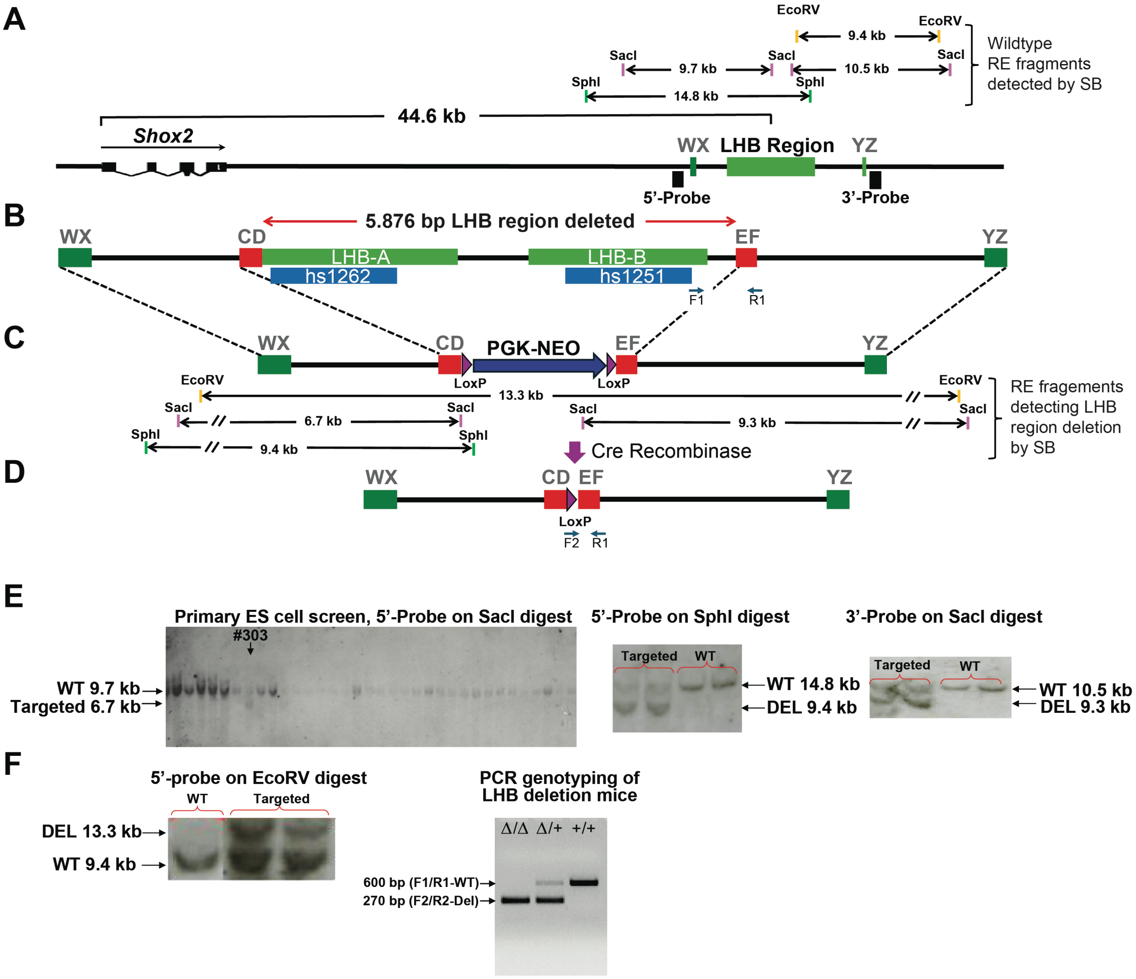

Supplementary Figure 7. Generation of mice harboring a genomic deletion of the LHB (hs1262-hs1251) region. (**A**) Map of 63 kb of the mouse *Shox2* genomic neighborhood, including the LHB region, which is centered 44.6 kb downstream of the *Shox2* TSS. The position of the WX and YZ sequences used to clone the LHB region, the location of relevant restriction enzyme (RE) fragments used for Southern blotting (SB), and the sequences serving as 5' and 3' external SB probes, are indicated. (**B**) Enlarged map of the WX to YZ, 11,978 bp genomic fragment retrieved from BAC RP23-213a24 by gap repair with enhancer locations shown. The 5,876 LHB region deleted in subsequent steps is flanked by the CD and EF sequences. (**C**) The genomic targeting construct for deletion of the LHB region. Recombineering in E. coli was used to delete the sequence flanked by the CD-EF sequences from the retrieved WX-YZ genomic fragment. This construct was then electroporated into G4 ES cells for genomic deletion of the LHB. Position and size of RE fragments used to detect the genomic deletion are indicated. (**D**) Structure of the final LHB-deleted (LHB$^{\Delta}$) allele generated by the *in vivo* excision of the neomycin selection cassette following transgenic expression of Cre recombinase. (**E**) SB screening for LHB genomic deletion in ES cell clones. Left: primary SB screening of a subset of the 358 ES cell clones, using an external 5' probe of SacI digests of genomic DNA. A single heterozygous targeted clone was identified (clone #303) by the presence of the 6.7 kb band specific for LHB deletion. Right: clone #303 genomic DNA and a WT control confirm heterozygous LHB deletion with external 5' and 3' probes. (**F**) Left: SB of genomic DNA confirms heterozygous LHB deletion in progeny of chimeric mice generated from clone #303 ES cells. Right: PCR genotyping for wildtype, heterozygous and LHB deletion mice

carrying the allele as shown in (D). The genomic locations of LHB PCR primer sequences F1/R1 (wild type) and F2/R1 (LHB deletion) are shown in (B) and (D), respectively.

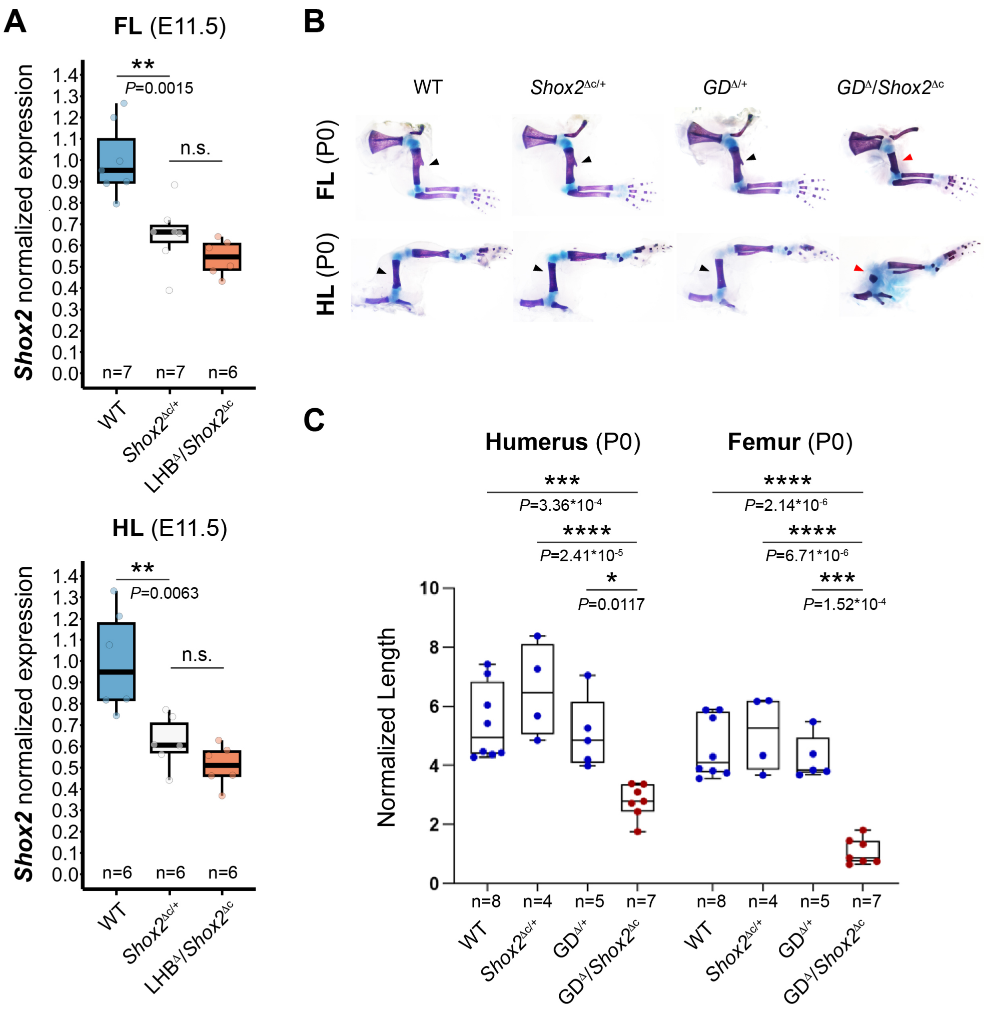

Supplementary Figure 8. Requirement of the gene desert for proximal limb development. (**A**) *Shox2* expression by qPCR in fore- (FL) and hindlimbs (HL) of wildtype (WT), *Shox2*$^{\Delta c/+}$ and LHB$^{\Delta}$/*Shox2*$^{\Delta c}$ embryos at E11.5. (**B**) Limb skeletons from control (*Shox2*$^{floxed/+}$ [WT], *Shox2*$^{\Delta c/+}$, GD$^{\Delta/+}$) and sensitized gene desert knockout (GD$^{\Delta}$/*Shox2*$^{\Delta c}$) mice at P0 are shown. Stylopod elements in fore- and hindlimbs of GD$^{\Delta}$/*Shox2*$^{\Delta c}$ newborns are reduced in length (red arrowheads) compared to normal stylopod morphology in controls (black arrowheads). Chondrogenic skeletal elements are stained blue, ossified structures red. (**C**) Quantification of ossified stylopod compartments at P0 reveals a significant reduction of humerus and femur length in GD$^{\Delta}$/*Shox2*$^{\Delta c}$ mice compared to WT, *Shox2*$^{\Delta c/+}$ and GD$^{\Delta/+}$ controls, respectively. Measurements were normalized to the length of the third metatarsal. Box plot indicates interquartile range, median, and maximum/minimum values (bars). Dots represent individual data points. ****, P ≤ 0.0001; ***, P ≤0.001; *, P ≤0.05 (one-way ANOVA). Source data are provided as a Source Data file.

Supplementary Tables.

Supplementary Table 1. Primers used for PCR amplification of predicted desert enhancer (DE) elements for Hsp68-*LacZ* reporter assays. Distance (in *kb*) from the *Shox2* TSS is indicated in brackets for each element (-, upstream; +, downstream).

Element ID	Forward primer	Reverse primer	Product size (bp)	Genomic coordinates (mm10)
**DE1 (+183)** (mm1849)	TCCAACTAGCCACAATCCACTA	GGTTGACAAAGGTTCAGAAAGG	2486	chr3 66797249 66799734
**DE2 (+297)** (mm1852)	TCCTCTCTGTGTTTCAGCTTTG	TGGGTGACTCAGGTAAACCTCT	3167	chr3 66682968 66686134
**DE3 (+330)** (mm1853)	ACCATGGTAGGAAGTTCATTGG	GTTAGAGCTGTTGGGAAAATGC	4408	chr3 66650021 66654428
**DE4 (+407)** (mm1837)	GCTATACGCCGTCAGCTTTAGT	ATGTGAATGAAGCACAAATTGC	3236	chr3 66572737 66575972
**DE5 (+437)** (mm2108)	GATGTGGGGAAACTTCTGAAAC	TACAGACCCAGACAAGAGCAGA	4335	chr3 66542058 66546392
**DE6 (+444)** (mm1845)	GATGCAGGCACGATATACAAAA	AGACCTTACACACGTGCACAAC	2962	chr3 66535471 66538432
**DE7 (+463)** (mm2103)	CTGCGCTTTCTTCTTATCCCTA	CAGATCCACCTCTTCCTTCATC	3402	chr3 66518263 66521664
**DE8 (+466)** (mm1838)	GGAATTGCTTTGTAGCTCTGCT	CAGGGAGGAAGCTTCTAGTTCA	1816	chr3 66514951 66516766
**DE9 (+475)** (mm1846)	GACACCACCAAGAGTTCGTGTA	AATTACAATGTGTGGGGGAGAC	2824	chr3 66504602 66507425
**DE10 (+487)** (mm2109)	TCTCTATGACCAAACGGGCTAT	GGATTTGGAAGAACAAGAGGTG	3109	chr3 66492941 66496049
**DE11 (+495)** (mm2110)	CTGTGTATGCCTTTGCTCTCAG	CTCTGCTCATATTCTGCCTCCT	3067	chr3 66484145 66487211
**DE12 (+501)** (mm1839)	CTGCTCTAATTCTGGGAGGTTG	TTATTGCTTGGTGAGAATGTGG	3041	chr3 66478804 66481844
**DE13 (+581)** (mm1842)	TGTATTCCACAGCCTCCCTAGT	CCCAAGGTCTGGTTTAGAACTG	2511	chr3 66400179 66402689
**DE14 (+583)** (mm2111)	TCCTACAGGCAAGACCTCTCTC	CATGGTCCAACTGGTATTGATG	1942	chr3 66397740 66399681
**DE15 (+606)** (mm1843)	CATTGGTACTTGGGCTGAAAA	TTACAAAGCTCCTGACGCAGT	3139	chr3 66373217 66376355
**DE16 (+656)** (mm2112)	CAGAGGTCCTGAACTCAATTCC	TCCTGCTGTGCATAGAACAACT	2839	chr3 66324764 66327602

Supplementary Table 2. CRISPR deletions and sgRNA templates. Genomic coordinates of the CRISPR-deleted regions are provided for each founder mouse line. For the gene desert deletion, use of unique sgRNAs resulted in the generation of two nearly identical founder lines (see Supplementary Fig. 4A, B).

**Mouse allele**	**Genomic coordinates of deletion** (mm10)	**Deleted region** (bp)	**5' sgRNA target sequence**	**5' sgRNA target sequence**
**GD$^{\Delta}$** (Founder 1)	chr3 66365062 66947168	582107	TGATCTTCATAACTGCCATGGGG	TGAAGCACAAGGCTGGCGGGAGG
**GD$^{\Delta}$** (Founder 2)	chr3 66365069 66947161	582093	TGATCTTCATAACTGCCATGGGG	TGAAGCACAAGGCTGGCGGGAGG
**SV-Enh$^{\Delta}$**	chr3 66654441 66658882	4442	GGGATACATTGAGACCGGCA	ACAGCAGTATCTGCCGTAGA

Supplementary Table 3. Primers used for screening and genotyping of CRISPR deletion mouse strains. PCR genotyping strategy and results using agarose gel electrophoresis are shown in Supplementary Figs. 4A, B (for the gene desert deletion) and 5E, F (for +325 SV enhancer deletion). GD, gene desert. Del, deletion. Enh, enhancer. P, primer pair. f, founder. N.A., not amplified.

Analyzed Region	Primer ID	Sequence	Product size (bp)
**GD-del (P1)**	**F1**	agcggagggatactttagcac	WT: 582587 (N.A.)
	**R1**	tgctgagagatgaaccctgat	KO: 480 (f1) / 494 (f2)
**GD-del 5’ junction (P2)**	**F2**	ccgcagagttctttgagagttt	WT: 611
	**R2**	gacccagcagtattcggagtta	KO: N.A.
**GD-del (P3)**	**F3**	ccgcagagttctttgagagttt	WT: 582603 (N.A.)
	**R3**	acaagagcatgtgttcaagtgg	KO: 496 (f1) / 510 (f2)
**GD-del 3’ junction (P4)**	**F4**	tgccctacagaagttaagcaca	WT: 455
	**R4**	tactgttgccatcactccattc	KO: N.A.
**Region 44 (+466kb)**	**44 F**	ggaattgctttgtagctctgct	WT: 1816
	**44 R**	cagggaggaagcttctagttca	KO: N.A.
**Region 40 (+389kb)**	**40 F**	tctataacggagctgcacttga	WT: 3308
	**40 R**	ggcatttgtgagacatgagaaa	KO: N.A.
**SV-Enh-del (P5)**	**F5**	ccaggataggaagaaagcaaga	WT: 4873 (N.A.)
	**R5**	agcaaggaggacaccaaagtag	KO: 431
**SV-Enh 5’ junction (P6)**	**F5**	ccaggataggaagaaagcaaga	WT: 329
	**R6**	taaagctagtccgtctcgtgtg	KO: N.A.
**Control region 1 (Ctrl-1)**	**Ctrl-1 F**	ccctagttctgtaaaccaggcta	WT/KO: 800
	**Ctrl-1 R**	tcatgtgtcttaggagagggttc	(Tbx3 locus)
**Control region 2 (Ctrl-2)**	**Ctrl-2 F**	agctggtagccttaaaataagccaa	WT/KO: 543
	**Ctrl-2 R**	gcctgaaagaggtcatcatcacc	(Gli3 locus)
**Control region 3 (Ctrl-3)**	**Ctrl-3 F**	ggaatgcccaggacataaaa	WT/KO: 172
	**Ctrl-3 R**	gagaagggcttatttcagctcac	(Gata4 locus)

Supplementary Table 4. Primers used for SYBR Green Real-time PCR analysis.

Target Gene	qPCR primer	Sequence	*Product size (bp)*
Shox2	Shox2_F	CCCGAGTACAGGTTTGGTTTC	119*
	Shox2_R	GGGTGCAACTCTACAAGCTTC	
Rsrc1	Rsrc1_F	TGCAATTGGTCCTTGAAGCT	104*
	Rsrc1_R	GGTGGCTTGGTCTTCTTCTT	
Actb	Actb_F	ACACTGTGCCCATCTACGAGG	280*
	Actb_R	CGCTCGTTGCCAATAGTGATG	

*primer pair validated previously[15].

Supplementary Table 5. Genomic elements tested via transgenic LacZ reporter assays based on cardiac ATAC-seq enhancer predictions. All elements were validated using conventional random transgenesis based on Hsp68-LacZ reporter constructs[17], except for the +325 element which was analyzed using site-directed transgenesis at the *H11* safe-harbor locus based on a targeting vector encoding a LacZ reporter unit with a minimal β-globin promoter (enSERT)[18,19]. Distance (in *kb*) from the *Shox2* TSS and Vista IDs are indicated in brackets for each element (-, upstream; +, downstream).

Element ID	*Forward primer*	*Reverse primer*	*Product size (bp)*	*Genomic coordinates (mm10)*
**+319** (mm2105)	GGTCAGGAATTCAGAGGTCAAC	ATACATCTGGGTTTGTCCATCC	3463	chr3 66660575 66664037
**+325** (mm2323)	ATCAGCTCAGCTTTGGTTAAGG	ACTGACCCTTCACAGACTGGTT	3982	chr3 66654695 66658676
**+325-A** (mm2106)	GCCATTATGGTCTTGAAGGAAG	ACTGACCCTTCACAGACTGGTT	3029	chr3 66655648 66658676
**+325-B** (mm2114)	ATCAGCTCAGCTTTGGTTAAGG	GAATTCCTGATGCACTCTTTCC	2502	chr3 66654695 66657196
**+389** (mm2099)	TCTATAACGGAGCTGCACTTGA	GGCATTTGTGAGACATGAGAAA	3307	chr3 66590716 66594023
**+405** (mm2102)	GATGTGGGGAAACTTCTGAAAC	TACAGACCCAGACAAGAGCAGA	3036	chr3 66575695 66578731
**+417** (mm2101)	CTGCCATAACATTTGTGCTGTT	AATGCTTGTTTCCCAGAAGGTA	3540	chr3 66562316 66565856
**+515** (m2100)	GGTTGACACAAGTAACCAGCAA	GCAAGCACTCTACCCCATAATC	3335	chr3 66465121 66468456
**+520** (mm2115)	GTATGTTGTGGGCTTTCTCCTC	ATGAATCCCATGTAAGCAAACC	3846	chr3 66459872 66463718

Supplementary Table 6. Viewpoints and primers used for 4C-Seq.

Viewpoint	Genomic coordinates (NlaIII fragment) (mm10)	Primer sequence
Shox2	chr3:66,980,317-66,981,259*	Forward/Reading primer**: *AATGATACGGCGACCACCGAACACTCTTTCCCTACACGACGCTCTTCCGATCT* **CCAATTAAGAAAATATGTGGCATG**<br><br>Reverse Primer: *CAAGCAGAAGACGGCATACGA***AGAATGTGAAGTTTGGTCCC**
**PLE2**	chr3:66,938,480-66,939,521	Forward/Reading primer: *AATGATACGGCGACCACCGAACACTCTTTCCCTACACGACGCTCTTCCGATCT* **ACTGCTTAGTAAAGACTAATTATTCATG**<br><br>Reverse Primer: *CAAGCAGAAGACGGCATACGA***AATGACATTATTATAAAATGCAATACTCT**
**PLE4**	chr3:66,573,586-66,574,775	Forward/Reading primer: *AATGATACGGCGACCACCGAACACTCTTTCCCTACACGACGCTCTTCCGATCT* **GGCTGATTCTCCTGCATG**<br><br>Reverse Primer: *CAAGCAGAAGACGGCATACGA***AGTTATAAAGATGATTAAGCTCTGATC**

*The *Shox2* viewpoint spans the 3' end of the first *Shox2* exon and the 5' end of intron 1.
** Illumina adapter sequences are shown in italics and sequence specific to the viewpoint in bold.

Supplementary Table 7. Proximal limb enhancers (PLEs) identified via 4C-seq and validated by β-globin promoter LacZ transgenesis. Distance (in *kb*) from the *Shox2* TSS is indicated in brackets for each element (-, upstream; +, downstream).

Element ID	*Forward primer*	*Reverse primer*	*Product size (bp)*	*Genomic coordinates (mm10)*
**PLE1 (-89)**	TGGGCAAAGATCACAGAACA	GTGTGTGTGTGTGTGGTGGA	1674	chr3 67070163 67071836
**PLE2 (+43)**	GAAGGACCGCACAGCTTATC	GGTCCACATATGCCCAAGGA	2428	chr3 66937659 66940086
**PLE3 (+237)**	GAAGAGGGGGCAGATTGTGTTGACTG	TGCTTCTTCAAATATTGCTTTGCTAAT	10351*	chr3 66739935 66750285
**PLE4 (+407)****	GTGAATGAAGCACAAATTGCAA	AAAGCCCATGTGTTCATCCCAG	3718	chr3 66572253 66575970
**PLE5 (+568)**	GGTCTATCTTGTTGCATGTTTTGTT	GGACAAACAGAGCTCAGAAGAGA	9473***	chr3 66409729 66419201

*A 9128bp ApaI/SalI sub-fragment (mm10: chr3:66740432-66749559) of the 10351bp PCR fragment was cloned into the p*βlacz* vector and used for LacZ transgenesis.
**The PLE4 fragment contains the DE4 element (Supplementary Table 1) and an additional 486bp.
***A 8520bp ApaI/SalI sub-fragment (mm10: chr3:66409729-66418248) of the 9473bp PCR fragment was cloned into the p*βlacz* vector and used for LacZ transgenesis.

Supplementary Table 8. PCR primers used for recombineering, Southern Blot (SB) probe amplification and genotyping of the mouse LHB deletion allele. Genomic locations of amplified products are shown in Supplementary Fig. 7. Nucleotides in *italics* represent restriction enzyme sites.

PCR fragment	***Primer name***	***Sequence***	***Product size (bp)***
**WX recombineering arm**	LHBW	ATAA*GCGGCCGC*AGATAAGAAGCAGCATAGAGGGC	360 bp
	LHBX	GTC*AAGCTT*TGGAGTATTTTGTGTGGGAGC	
**YZ recombineering arm**	LHBY	GTC*AAGCTT*CCTCACGACTACTGCCCATTTC	245 bp
	LHBZ	TCT*ACTAGT*CCAGCAAGACCACATCACCTC	
**CD recombineering arm**	LHBC	ATAA*GCGGCCGC*AAAGAACCCAGTCTTGTGGAATT	262 bp
	LHBD	GTC*GAATTCGAGCTC*TTTGCTTCTGTGAATCCTTTTCTAC	
**EF recombineering arm**	LHBE	ATAG*GATCC*TGGTTTGTAGGGTGGTAAAAATGC	293 bp
	LHBF	GTC*GTCGAC*AGTTTGGTGGCAAGAATCCTTC	
**5' External Probe for SB**	5PROBER	CAAGGACAAAGCCCCTATTTTTC	731 bp
	5PROBER	GTTATACTGGGAGAAAGGGCGAT	
**3' External Probe for SB**	3PROBEF	TCCTCTGCCTTGAGCTTGGG	750 bp
	3PROBER	ATGAGTCGAGGTATGAAAAATCGT	
	LHBF1	AAAATATGCGTTGGTGGGAA	WT: 600 bp
**LHB$^{\Delta}$ genotyping**	LHBF2	GCCCAATTCCGATCATATTC	Del: 270 bp
	LHBR1	CCATTTCTTGCTTCTAAGTCCCTG	

SUPPLEMENTARY REFERENCES

1. Dixon, J. R. *et al.* Topological domains in mammalian genomes identified by analysis of chromatin interactions. *Nature* **485**, 376–380 (2012).
2. Gorkin, D. U. *et al.* An atlas of dynamic chromatin landscapes in mouse fetal development. *Nature* **583**, 744–751 (2020).
3. Bonev, B. *et al.* Multiscale 3D Genome Rewiring during Mouse Neural Development. *Cell* **171**, 557-572.e24 (2017).
4. ENCODE Project Consortium *et al.* Expanded encyclopaedias of DNA elements in the human and mouse genomes. *Nature* **583**, 699–710 (2020).
5. Justice, M., Carico, Z. M., Stefan, H. C. & Dowen, J. M. A WIZ/Cohesin/CTCF Complex Anchors DNA Loops to Define Gene Expression and Cell Identity. *Cell Rep.* **31**, 107503 (2020).
6. Mifsud, B. *et al.* GOTHiC, a probabilistic model to resolve complex biases and to identify real interactions in Hi-C data. *PLoS One* **12**, e0174744 (2017).
7. Blaschke, R. J. *et al.* Targeted mutation reveals essential functions of the homeodomain transcription factor Shox2 in sinoatrial and pacemaking development. *Circulation* **115**, 1830–1838 (2007).
8. Akerberg, B. N. *et al.* A reference map of murine cardiac transcription factor chromatin occupancy identifies dynamic and conserved enhancers. *Nat. Commun.* **10**, 4907 (2019).
9. He, A. *et al.* Dynamic GATA4 enhancers shape the chromatin landscape central to heart development and disease. *Nat. Commun.* **5**, 4907 (2014).
10. Liang, X. *et al.* Transcription factor ISL1 is essential for pacemaker development and function. *J. Clin. Invest.* **125**, 3256–3268 (2015).
11. van Eif, V. W. W. *et al.* Genome-Wide Analysis Identifies an Essential Human TBX3 Pacemaker Enhancer. *Circ. Res.* **127**, 1522–1535 (2020).
12. Jolma, A. *et al.* DNA-binding specificities of human transcription factors. *Cell* **152**, 327–339 (2013).

13. Chen, S., Lee, B., Lee, A. Y.-F., Modzelewski, A. J. & He, L. Highly Efficient Mouse Genome Editing by CRISPR Ribonucleoprotein Electroporation of Zygotes. *J. Biol. Chem.* **291**, 14457–14467 (2016).

14. Ye, W. *et al.* A unique stylopod patterning mechanism by Shox2-controlled osteogenesis. *Development* **143**, 2548–2560 (2016).

15. Osterwalder, M. *et al.* Enhancer redundancy provides phenotypic robustness in mammalian development. *Nature* **554**, 239–243 (2018).

16. Rodríguez-Carballo, E., Lopez-Delisle, L., Yakushiji-Kaminatsui, N., Ullate-Agote, A. & Duboule, D. Impact of genome architecture on the functional activation and repression of Hox regulatory landscapes. *BMC Biol.* **17**, 55 (2019).

17. Osterwalder, M. *et al.* Characterization of Mammalian In Vivo Enhancers Using Mouse Transgenesis and CRISPR Genome Editing. *Methods Mol. Biol.* **2403**, 147–186 (2022).

18. Kvon, E. Z. *et al.* Comprehensive In Vivo Interrogation Reveals Phenotypic Impact of Human Enhancer Variants. *Cell* **180**, 1262-1271.e15 (2020).

19. Darbellay, F. *et al.* Pre-hypertrophic chondrogenic enhancer landscape of limb and axial skeleton development. *Nat. Commun.* **15**, 4820 (2024).
