# Supplementary material for: A gene desert required for regulatory control of pleiotropic Shox2 expression and embryonic survival
Source: Nat Commun. 2024 Oct 10;15:8793. doi: 10.1038/s41467-024-53009-7 (PMC11467299; doi:10.1038/s41467-024-53009-7)
Supplement: Supplementary file 3 — Description of additional supplementary files [file 41467_2024_53009_MOESM3_ESM.pdf]

Description of Additional Supplementary Files

Supplementary Data 1. Developmental enhancer predictions within the *Shox2* TAD. Sheet 1: List of genomic elements within the extended *Shox2* TAD interval (chr3:65996078-67396078) that show significant ENCODE H3K27ac ChIP-seq enrichment in at least one tissue at any developmental stage and that were further filtered for ChromHMM[1] strong enhancers calls (as defined in Gorkin *et al.*, 2020[2]). H3K27ac RPKM (Reads Per Kilobase of transcript, per Million mapped reads) values are shown for each developmental tissue and timepoint in a matrix format and are underlying the heatmap shown in Fig. 1B and Supplementary Fig. 1A. For genes present in this domain, RNA-seq read counts are listed as fragments per kilobase of exon per million reads mapped (FPKM) (red shaded). Blue shades mark predicted gene desert enhancers (DEs). Mouse (mm10) coordinates (chrom, start, end) are given for each putative enhancer identified. Element IDs indicate distance to *Shox2* transcriptional start site (TSS). Sheet 2: The same matrix as in *sheet 1* but including q-values (-log10(q)) (minimum FDR) for each H3K27ac-enriched region as a measurement of statistical significance. Sheet 3: Metadata to be able to retrieve peak lists (for q-values) from the ENCODE Data Coordination Center (DCC, http://www.encodeproject.org/). Sheet 4: More permissive set of putative tissue-specific enhancers within the *Shox2* TAD and in major *Shox2* expressing tissues, based on peak calls with less stringent parameters and addition of the E10.5 dataset. H3K27ac-thresholds were applied from more to less stringent: p-value<0.00001, q-value<0.05, p-value<0.001 (q: minimum FDR, p: Bejnamini-Hochberg correction). "X" (blue field) denotes a H3K27ac peak call. Element ID lists regions called with stringent predictions (*Sheet 1*). Coordinates are mm10 and gene IDs are listed in red. Sheet 5: More permissive set of putative tissue-specific enhancers (as in *Sheet 4*) but filtered for strong TSS-distal enhancer elements using ChromHMM. "X" (green field) denotes a filtered H3K27ac peak call. Element ID lists regions called with stringent predictions (Sheet 1). Coordinates are mm10 and gene IDs are listed in red.

Supplementary Data 2. C-HiC domain boundaries and *Shox2*-interacting regions by GOTHiC. Sheet 1: Genomic coordinates (mm10) of inter-domain boundaries, related p-values and TAD separation scores (TAD_SEP) resulting from HiCExplorer[3] analysis of *Shox2* C-HiC datasets (interrogated interval: chr3:65196078-68696078). $P<10^{-12}$ is listed as "0" (Wilcoxon rank-sum test). FL, forelimb. MD, mandible. HT, Heart. Sheets 2-4: Genomic coordinates (mm10) of elements showing significant Hi-C contacts with *Shox2* as determined by GOTHiC[4] using a 10kb viewpoint on the *Shox2* TSS in forelimb (FL), mandible (MD) or heart (HT) tissues (interrogated interval: chr3:65196078-68696078), respectively. Sheets 5-7: Genomic coordinates (mm10) of elements showing significant Hi-C contacts with the SV-enhancer as determined by GOTHiC[4] using a 4kb viewpoint on the SV-enhancer in forelimb (FL), mandible (MD) or heart (HT) tissues (interrogated interval: chr3:65196078-68696078). Q-values in sheets 2-7 are determined by cumulative binomial test (GOTHiC).

Supplementary Data 3. Reprocessed ATAC-seq and ChIP-seq datasets used in this study. List of previously published mouse ATAC-seq and ChIP-seq datasets from embryonic hearts (E12.5), sinoatrial node (SAN, P0) and limb re-processed as part of this study using uniform pipelines (see Methods). The resulting peak datasets (r: replicate; int: intersected) are listed and provided as NarrowPeak files in Supplementary Data 5. FL, forelimb. HL, hindlimb. r, replicate.

Supplementary Data 4. List of PWMs from heart-expressed TFs for genome-wide cardiac TF motif prediction. Of the 1'376 motifs obtained, 14 (shaded in gray) were part of larger motifs and thus omitted in the analysis (see Methods).

Supplementary Data 5. NarrowPeak files of reprocessed ATAC-seq and ChIP-seq datasets (as listed in Supplementary Data 3**).**
