# Supplementary material for: A gene desert required for regulatory control of pleiotropic Shox2 expression and embryonic survival
Source: Nat Commun. 2024 Oct 10;15:8793. doi: 10.1038/s41467-024-53009-7 (PMC11467299; doi:10.1038/s41467-024-53009-7)
Supplement: Supplementary file 9 — Reporting Summary [file 41467_2024_53009_MOESM9_ESM.pdf]

nature portfolio

Corresponding author(s): Marco Osterwalder, John Cobb

Last updated by author(s): Aug 30, 2024

Reporting Summary

Nature Portfolio wishes to improve the reproducibility of the work that we publish. This form provides structure for consistency and transparency in reporting. For further information on Nature Portfolio policies, see our Editorial Policies and the Editorial Policy Checklist.

Statistics

For all statistical analyses, confirm that the following items are present in the figure legend, table legend, main text, or Methods section.

n/a	Confirmed	
☐	☒	The exact sample size (*n*) for each experimental group/condition, given as a discrete number and unit of measurement
☒	☐	A statement on whether measurements were taken from distinct samples or whether the same sample was measured repeatedly
☐	☒	The statistical test(s) used AND whether they are one- or two-sided<br>*Only common tests should be described solely by name; describe more complex techniques in the Methods section.*
☒	☐	A description of all covariates tested
☒	☐	A description of any assumptions or corrections, such as tests of normality and adjustment for multiple comparisons
☐	☒	A full description of the statistical parameters including central tendency (e.g. means) or other basic estimates (e.g. regression coefficient) AND variation (e.g. standard deviation) or associated estimates of uncertainty (e.g. confidence intervals)
☐	☒	For null hypothesis testing, the test statistic (e.g. *F*, *t*, *r*) with confidence intervals, effect sizes, degrees of freedom and *P* value noted<br>*Give P values as exact values whenever suitable.*
☒	☐	For Bayesian analysis, information on the choice of priors and Markov chain Monte Carlo settings
☒	☐	For hierarchical and complex designs, identification of the appropriate level for tests and full reporting of outcomes
☒	☐	Estimates of effect sizes (e.g. Cohen's *d*, Pearson's *r*), indicating how they were calculated

Our web collection on statistics for biologists contains articles on many of the points above.

Software and code

Policy information about availability of computer code

Data collection	No commercial, open source or custom software or code were used for data collection in this study.
Data analysis	For C-HiC, paired-end reads were mapped to the NCBI38/mm10 reference genome using Bowtie2 (v2.4.5) (Langemad and Salzberg 2012) and further filtered and de-duplicated using HiCUP (v0.8.1). Valid and unique di-tags were filtered and processed using Juicer command line tools (v1.9.9) to generate binned contact maps from valid read pairs with MAPQ≥30. The hicConvertFormat tool (HiCExplorer v3.7.2) was used to generate raw .cool files from native .hic out-puts generated by Juicer. The Cooler matrix balancing tool (v0.8.11) was applied for normalization and diagonal filtering. HiCExplorer (v3.7.2) was used to determine normalized inter-domain insulation scores and domain boundaries. GOTHiC (v.1.32.0) was used to identify significance-based Hi-C interactions from HiCUP validated read pairs. For Virtual 4C, peak profiles were generated using custom Java code based on htsjdk v2.12.0 and the BigwigCompare tool (deepTools v3.5.1) was used to generate relative subtraction profiles.<br>For mouse ATAC-seq and ChIP-seq (re-)analysis Cutadapt was used for adaptor trimming (trim_galore_v0.6.6) and Bowtie2 (v2.4.2) was used for read mapping. Duplicates and low-quality reads were removed using SAMtools (v1.12 or v1.14). Peak calling was performed using MACS2 (v2.1.0 or v2.2.7.1). For ENCODE ChIP-seq analysis Bowtie was used for alignment to the genome and peak calling was performed using MACS (v1.4) instead. Evidence from two biological replicates was combined using IDR (https://www.encodeproject.org/data-standards/terms/). For extended ENCODE-based predictions isogenic replicates were concatenated and further merged using bedtools (v2.30.0). Peaks were extracted using the BEDOPS tool (v2.4.39). GREAT (v4.0.4) was used to evaluate TSS proximity.<br>For ChIP-seq and RNA-seq analysis from human fetal hearts cutadapt (v1.1) was used for quality filtering and adaptor trimming. For ChIP-seq Bowtie (v2.0.2.0) was used for read mapping and peak calling and duplicates were removed using SAMtools. Tophat v2.0.6 was used to align RNA-seq reads and reads mapping to UCSC known genes (hg19) were determined by HTSeq (v0.7.0).<br>For cardiac TF motif detection the BiomaRt v2.5.0 package (R v4.1.2) was used for mapping of gene symbols to identifiers. FIMO v5.3.0 was used for detection of TF-binding sites and BWTOOL v1.0 was used for computation of motif conservation based on conservation scores

provided by the UCSC PHAST package.
For 4C-Seq, de-multiplexed reads were mapped to mouse genome assembly GRCm38/mm10 using Bowtie and analyzed as reads per NlaIII restriction fragment within the 4C-seq module of the HTSstation pipeline (David et al., 2014). Within HTSstation, interaction profiles were normalized to a 5 Mb genomic region surrounding the viewpoint and smoothened using a window size of 11 fragments.
Brightness and contrast of acquired embryo images were adjusted uniformly using Photoshop (CS5 or v22).
For micro-CT scans, NRecon (v1.7.4.2) was used to perform stack reconstructions and 3D landmarks were placed using MeshLab (v2020.07).
µCT measurement plots were generated and analyzed using GraphPad Prism (v10.2.3). All other statistics were estimated, and plots were generated using the statistical computing environment R version 4.3.2.

For manuscripts utilizing custom algorithms or software that are central to the research but not yet described in published literature, software must be made available to editors and reviewers. We strongly encourage code deposition in a community repository (e.g. GitHub). See the Nature Portfolio guidelines for submitting code & software for further information.

Data

Policy information about availability of data

All manuscripts must include a data availability statement. This statement should provide the following information, where applicable:

- Accession codes, unique identifiers, or web links for publicly available datasets
- A description of any restrictions on data availability
- For clinical datasets or third party data, please ensure that the statement adheres to our policy

The raw and processed next-generation sequencing (NGS) datasets generated in this study have been deposited in the NCBI GEO database under accession codes GSE161194 (4C-seq) and GSE232887 (super-series including C-HiC (GSM7385429-30), ATAC-seq (GSM7385432-33), ChIP-seq (GSM7385434-41) and RNA-seq data (GSM7385442-45)). Accession codes of previously published ATAC-seq (GSE124338, GSE148515, GSE126293) and ChIP-seq (GSE96107; GSE137285; GSE124008, GSE52123, GSE68974, GSE123388, GSE129427; ENCODE: ENCFF310VOQ, ENCFF464DYI) datasets reprocessed in this study are listed in Supplementary Data 3 with the respective NarrowPeak files are available in Supplementary Data 5. Wherever applicable, reference genomes Mouse GRCm38/mm10 and Human GRCh37/hg19 were used for alignment and comparisons. Images of transgenic embryos with LacZ-reporter activity are available at the Vista Enhancer Browser (http://enhancer.lbl.gov). Source data are provided with this paper. Correspondence and requests for materials should be addressed to J.C. (jacobb@ucalgary.ca) or M.O. (marco.osterwalder@unibe.ch).

Research involving human participants, their data, or biological material

Policy information about studies with human participants or human data. See also policy information about sex, gender (identity/presentation), and sexual orientation and race, ethnicity and racism.

Reporting on sex and gender	Heart tissue samples of cardiac compartments (LV, LA, RV, RA) from only one human embryo (XY) at post conception week 17 were analyzed. Sex as a variable in our studies at fetal stages of heart development are expected to show minimal differences.
Reporting on race, ethnicity, or other socially relevant groupings	n/a
Population characteristics	PCW17_12570 46, XY
Recruitment	n/a
Ethics oversight	All aspects involving human tissue samples were reviewed and approved by the Human Subjects Committee at Lawrence Berkeley National Laboratory (LBNL) Protocol Nos. 00023126 and 00022756.<br>Fetal human heart samples were obtained from the Human Developmental Biology Resource's Newcastle site (HDBR, hdbr.org), in compliance with applicable state and federal laws. The National Research Ethics Service reviewed the HDBR study under REC Ref 23/NE/0135, and IRAS project ID: 330783 in compliance with requirements from the National Health Services for research within the UK and overseas. HDBR is a non-commercial entity funded by the Wellcome Trust and Medical Research Council. Fetal tissue donation is confidential, anonymized, completely voluntary with fully informed and explicitly documented written consent, and the participants do not receive compensation. In accordance, no identifying information for human samples in this study was shared by HDBR. More information about HDBR policies and ethical approvals can be accessed at https://www.hdbr.org/ethical-approvals.

Note that full information on the approval of the study protocol must also be provided in the manuscript.

Field-specific reporting

Please select the one below that is the best fit for your research. If you are not sure, read the appropriate sections before making your selection.

☒ Life sciences ☐ Behavioural & social sciences ☐ Ecological, evolutionary & environmental sciences

For a reference copy of the document with all sections, see nature.com/documents/nr-reporting-summary-flat.pdf

Life sciences study design

All studies must disclose on these points even when the disclosure is negative.

Sample size	Details are provided in the Methods section including specific information related to the experimental approach used.
Data exclusions	Mouse embryos were excluded from further analysis if the developmental stage was not correct or if they were not of the genotype of interest. No data exclusions except for well-established criteria in specific data filtering steps.
Replication	Experiments were conducted in replicates when the outcome was likely to have a significant impact on the interpretation of results.<br>For transient transgenic reporter analysis in mouse embryos, sample size selection and scoring criteria were based on our experience of performing transgenic mouse assays for >3,000 total putative enhancers (VISTA Enhancer Browser: http://enhancer.lbl.gov). Transgenic results were confirmed in at least n=3 (for Hsp68-LacZ or Beta-lacZ random integration) or n=2 (for enSERT) independent biological replicates while using criteria consistent with the pipeline established for the VISTA EnhancerBrowser (see Methods).<br>For genomic deletion (knockout) studies, sample sizes were selected empirically based on our previous studies and the standard in the field. All phenotypic characterization of knockout mice employed a matched littermate selection strategy. The minimal number of biological replicates analyzed per experiment is indicated in the respective Methods paragraphs. Briefly, for analysis of gene expression via ISH at least n=3 embryos (biological replicates) were analyzed per genotype. For immunofluorescence (IF) at least n=2 WT embryos and n=3 MUT embryos were analyzed. For quantative PCR (qPCR) analysis, respective tissues from at least n=5 embryos (biological replicates) were analyzed per genotype. For skeletal analysis at P0, fore- and hindlimbs of at least n=4 biological replicates were analyzed for control genotypes and at least n=7 for the GDd/Shox2dc genotype. For micro-CT analysis, fore- and hindlimb skeletons at P42 from at least n=4 biological replicates were measured for control genotypes and n=8 for the GDd/Shox2dc genotype.<br>For ATAC-seq from wildtype mouse embryonic hearts at E11.5 we analyzed n=2 biological replicate samples, each derived from a pool of n=2 micro-dissected hearts. For H3K27ac ChIP-seq and RNA-seq from human fetal cardiac compartments at pcw17, due to limited availability of human fetal heart samples from different cardiac compartments, left (LV) and right (RV) ventricular samples as well as left (LA) and right (RA) atrial samples were used to determine atrial and ventricular-specific signatures, respectively.<br>C-HiC datasets from limb, mandible and heart at E11.5 were processed in singlicate as the resulting matrices enable cross-comparison of contacts. For 4-C-seq studies n=2 independent biological replicate samples consisting of 10-12 pooled proximal mouse forelimbs at E12.5 were used.
Randomization	Unless for skeletal measurements, randomization was not required given the observational nature of the study and inclusion of appropriate biological replicates.
Blinding	Embryonic littermates and samples from genetically modified animals were dissected and processed blind to genotype. Individuals who qualitatively assessed the results of in vivo transgenic reporter assays or measured skeletal elements were blinded to genotyping information. For all other experiments, the investigators were not blinded to allocation during experiments and outcome assessment.

Reporting for specific materials, systems and methods

We require information from authors about some types of materials, experimental systems and methods used in many studies. Here, indicate whether each material, system or method listed is relevant to your study. If you are not sure if a list item applies to your research, read the appropriate section before selecting a response.

Materials & experimental systems

n/a	Involved in the study	
☐	☒	Antibodies
☒	☐	Eukaryotic cell lines
☒	☐	Palaeontology and archaeology
☐	☒	Animals and other organisms
☒	☐	Clinical data
☒	☐	Dual use research of concern
☒	☐	Plants

Methods

n/a	Involved in the study	
☐	☒	ChIP-seq
☒	☐	Flow cytometry
☒	☐	MRI-based neuroimaging

Antibodies

Antibodies used	Anti-SHOX2 (dilution: 1:300, Santa Cruz, # JK-6E, sc-81955)<br>Anti-SMA-Cy3 (1:250, Sigma, #C6198)<br>Anti-HCN4 (1:500, Thermo Fisher, #MA3-903)<br>Anti-NKX2.5 (1:500, Thermo Fisher, #PA5-81452)<br>Anti-H3K27ac (Active Motif, #39133)<br>Alexa Fluor 647 donkey anti-mouse (1:1000, Thermo Fisher, #A31571)<br>Alexa Fluor 568 goat anti-rat (1:1000, Thermo Fisher, #A11077)<br>Alexa Fluor 488 goat anti-rabbit (1:1000, Thermo Fisher, #A11008)
Validation	Antibodies used for IF were validated in mouse studies and exhibit the expected embryonic compartment- and/or cell type-specific

Validation	profiles. The anti-H3K27ac antibody was validated and used in previous studies for ChIP-seq in embryonic and fetal human tissues.

Animals and other research organisms

Policy information about studies involving animals; ARRIVE guidelines recommended for reporting animal research, and Sex and Gender in Research

Laboratory animals	Mice used for transient transgenic reporter analysis and mice of the GDd line (strain: FVB/NJ) were housed at the LBNL Animal Care Facility, which is fully accredited by AAALAC International. Stable transgenic reporter mouse lines (strain: CD-1) and mice of the GDd (strain: mixed FVB/C57BL/6NCrl) and LHBd (strain: C57BL/6NCrl) genomic deletion lines were housed at the Life and Environmental Sciences Animal Resource Centre at the University of Calgary accredited by the Canadian Council on Animal Care. Mice of the SV-Enhd line (strain: FVB/NRj) were housed at the Central Animal Facilities (CAF) of the Experimental Animal Center, University of Bern. The CAF runs upon approval of the Cantonal Authority, with husbandry license BE02/2022.<br>All mice were maintained with water supply on a 12:12 light-dark cycle, with relative humidity set at 30–70% (LBNL, University of Bern) or 20-50% (University of Calgary), and a temperature of 20–26.2 °C (LBNL, Calgary) or 22°C +/- 2°C (Bern). Mice at LBNL were housed in standard micro-isolator cages on hard-wood bedding with enrichment consisting of crinkle-cut naturalistic paper strands and fed on ad libitum PicoLab Rodent Diet 20 (5053). Mice at the University of Calgary were house in Tecniplast Blue Line IVC cages on hard-wood aspen chip bedding (autoclaved) with enrichment consisting of crinkle-cut naturalistic paper strands and a Cocoon nestlet (5800), while maintained on ad libitum irradiated PicoLab Mouse Diet 20 (5058). Mice at the University of Bern were housed in standard IVC cages GM500 Tecniplast, on Safe® Aspen wood granulate bedding with enrichment consisting of Pura® crinkle brown kraft paper nesting material, Pura® Brick Aspen Chew Block, and red polycarbonate mouse house and fed on Kliba Nafag standard breeding (3800) and maintenance diet (3430). All mice were health checked and monitored daily for food and water intake by trained personnel. Euthanasia at LBNL and University of Bern was performed in the home cage using CO2 asphyxiation while ensuring gradual fill and displacement rate. Euthanasia at University of Calgary was performed by cervical dislocation after loss of consciousness induced by isoflurane anesthesia administered by the bell-jar method (250µl on a gauze in a one liter container). Unless specified otherwise, mice between 6 to 30 (predominantly 6 to 10) weeks of age were used for breeding to generate embryos, newborns or adults analyzed in this study.
Wild animals	n/a
Reporting on sex	Animals of both sexes were used in these analyses. Sex was not considered as a variable in our embryonic studies since limb, craniofacial or heart development are expected to show minimal differences at the respective early stages of development. Skeletal analysis at P0 included both sexes as we did not expect normalized skeletal growth at P0 to show significant sex-based differences. Sex was tracked in mice used for micro-CT measurements at P42 and no significant sex-specific differences were observed after normalization.
Field-collected samples	n/a
Ethics oversight	All animal work at Lawrence Berkeley National Laboratory (LBNL, CA, USA) was reviewed and approved by the LBNL Animal Welfare Committee under protocol numbers 290003 and 290008. All animal work at the University of Calgary was reviewed and approved by the Life and Environmental Sciences Animal Care Committee (LESACC) under protocols AC21-0005 and AC21-0006, and in accordance with Canadian Council on Animal Care guidelines as approved by the University of Calgary (protocol AC13-0053). All animal work in Switzerland was reviewed and approved by the regional commission on Animal Experimentation and the Cantonal Veterinary Office of the city of Bern (protocol BE96/20).

Note that full information on the approval of the study protocol must also be provided in the manuscript.

Plants

Seed stocks	*Report on the source of all seed stocks or other plant material used. If applicable, state the seed stock centre and catalogue number. If plant specimens were collected from the field, describe the collection location, date and sampling procedures.*
Novel plant genotypes	*Describe the methods by which all novel plant genotypes were produced. This includes those generated by transgenic approaches, gene editing, chemical/radiation-based mutagenesis and hybridization. For transgenic lines, describe the transformation method, the number of independent lines analyzed and the generation upon which experiments were performed. For gene-edited lines, describe the editor used, the endogenous sequence targeted for editing, the targeting guide RNA sequence (if applicable) and how the editor was applied.*
Authentication	*Describe any authentication procedures for each seed stock used or novel genotype generated. Describe any experiments used to assess the effect of a mutation and, where applicable, how potential secondary effects (e.g. second site T-DNA insertions, mosiacism, off-target gene editing) were examined.*

ChIP-seq

Data deposition

☒ Confirm that both raw and final processed data have been deposited in a public database such as GEO.

☒ Confirm that you have deposited or provided access to graph files (e.g. BED files) for the called peaks.

Data access links<br>*May remain private before publication.*	https://www.ncbi.nlm.nih.gov/geo/<br>Accession: GSE232883

Files in database submission	LPCS_100915_1B_pcw17_12570_lv_h3k27ac.fastq.gz<br>LPCS_100915_1E_pcw17_12570_lv_input.fastq.gz<br>LPCS_100915_1A_pcw17_12570_la_h3k27ac.fastq.gz<br>LPCS_100915_1D_pcw17_12570_la_input.fastq.gz<br>LPCS_082815_2E_pcw17_12570_rv_h3k27ac.fastq.gz<br>LPCS_082815_2G_pcw17_12570_rv_input.fastq.gz<br>LPCS_082815_2D_pcw17_12570_ra_h3k27ac.fastq.gz<br>LPCS_082815_2F_pcw17_12570_ra_input.fastq.gz<br>pcw17_12570_lv_h3k27ac.hg19.uu.sort.ext.rpm.bw.gz<br>pcw17_12570_rv_h3k27ac.hg19.uu.sort.ext.rpm.bw.gz<br>pcw17_12570_la_h3k27ac.hg19.uu.sort.ext.rpm.bw.gz<br>pcw17_12570_ra_h3k27ac.hg19.uu.sort.ext.rpm.bw.gz<br>pcw17_12570_lv_h3k27ac_peaks.bed.gz<br>pcw17_12570_rv_h3k27ac_peaks.bed.gz<br>pcw17_12570_la_h3k27ac_peaks.bed.gz<br>pcw17_12570_ra_h3k27ac_peaks.bed.gz
Genome browser session<br>(e.g. UCSC)	https://genome.ucsc.edu/s/Marco%20Osterwalder/GSE232883

Methodology

Replicates	Due to limited availability of human fetal samples from different cardiac compartments, left (LV) and right (RV) ventricular samples as well as left (LA) and right (RA) atrial samples were used to determine atrial and ventricular-specific signatures, respectively.
Sequencing depth	Sample | Total Reads | Uniquely mapped reads | Read length | Read type<br>pcw17_12570_lv_h3k27ac | 28,505,380 | 25,222,464 | 50bp | single-end<br>pcw17_12570_la_h3k27ac | 24,883,304 | 22,092,838 | 50bp | single-end<br>pcw17_12570_rv_h3k27ac | 24,159,864 | 19,347,765 | 50bp | single-end<br>pcw17_12570_ra_h3k27ac | 20,763,585 | 14,985,826 | 50bp | single-end
Antibodies	Anti-H3K27ac antibody, Active Motif, Cat# 39133, Lot 01613007.
Peak calling parameters	MACS (v1.4.2) with parameter '-mfold = 10,30 -nomodel -p 0.0001' was used for peak calling.
Data quality	pcw17_12570_lv_h3k27ac | 46,379<br>pcw17_12570_la_h3k27ac | 51,591<br>pcw17_12570_rv_h3k27ac | 48,161<br>pcw17_12570_ra_h3k27ac | 46,628
Software	Reads were mapped to the human reference genome version hg19 using Bowtie (v2.0.2.0) with parameter '-m 1 -v 2 -p 16'.<br>Duplicates were removed using SAMtools.
